# γδ T Cells and Tumor Microenvironment: From Immunosurveillance to Tumor Evasion

**DOI:** 10.3389/fimmu.2018.01395

**Published:** 2018-06-15

**Authors:** Elena Lo Presti, Gabriele Pizzolato, Anna Maria Corsale, Nadia Caccamo, Guido Sireci, Francesco Dieli, Serena Meraviglia

**Affiliations:** ^1^Department of Biopathology, Università degli Studi di Palermo, Palermo, Italy; ^2^Department of Biomedical Sciences, Humanitas Università, Rozzano, Italy

**Keywords:** gamma delta T cells, tumor microenvironment, immunotherapy, cyototxicity, immunosuppression

## Abstract

γδ T cells possess cytotoxic antitumor activity mediated by production of proinflammatory cytokines, direct cytotoxic activity, and regulation of the biological functions of other cell types. Hence, these features have prompted the development of therapeutic strategies in which γδ T cells agonists or *ex vivo*-expanded γδ T cells are administered to tumor patients. Several studies have shown that γδ T cells are an important component of tumor-infiltrating lymphocytes in patients affected by different types of cancer and a recent analysis of ~18,000 transcriptomes from 39 human tumors identified tumor-infiltrating γδ T cells as the most significant favorable cancer-wide prognostic signature. However, the complex and intricate interactions between tumor cells, tumor microenvironment (TME), and tumor-infiltrating immune cells results in a balance between tumor-promoting and tumor-controlling effects, and γδ T cells functions are often diverted or impaired by immunosuppressive signals originating from the TME. This review focuses on the dangerous liason between γδ T cells and tumoral microenvironment and raises the possibility that strategies capable to reduce the immunosuppressive environment and increase the cytotoxic ability of γδ T cells may be the key factor to improve their utilization in tumor immunotherapy.

## The Tumor Microenvironment (TME)

Tumors develop in a composite and heterogeneous microenvironment consisting of endothelial cells, stromal cells, and immune cells; all of them act and cooperate either in direct or indirect way with tumor cells promoting tumor proliferation, invasion, and metastasis or actively interfering with its development.

It is well known that a large number of cells of both the innate and adaptive immune compartments are present at the tumor site since the early steps of cancer development, exerting immunosurveillance (1) and controlling spontaneous neoplastic diseases (2), even though the composition and extent of the immune infiltrates consistently varies among individuals (3, 4).

Tumors are able to escape from the host immune system and take advantage on the presence of infiltrating immune cells by modifying their functions and creating a TME favorable to tumor progression. In fact, tumor-infiltrating immune cells, together with stromal cells and extracellular matrix create an inflammatory milieu responsible for tumor expansion and dissemination and for tumor evasion. Tumor escape from the host immune response is promoted by its ability to actively subvert antitumor immunity by interfering with cell development, differentiation, migration, and cytotoxicity or from host immunosuppression.

**Figure 1:**
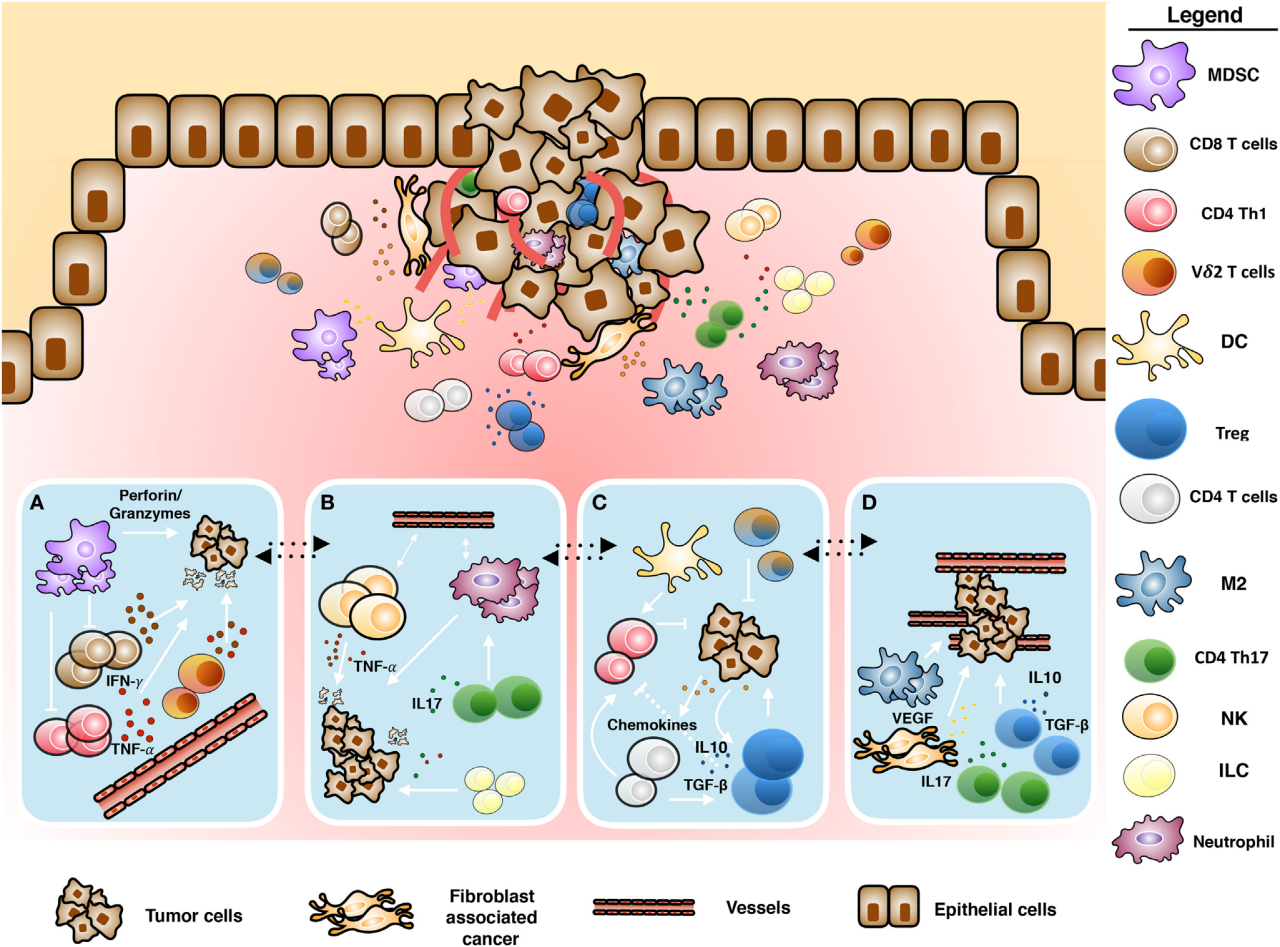
Schematic representation of tumor microenvironment. Cross-talk between immune and tumoral cells: **(A)** antitumoral role of infiltrating γδ T cells mediated by proinflammatory cytokines and cytotoxic activity, **(B)** inflammation induced by tumor-infiltrating immune cells, **(C)** immunosuppressive polarization of immune cells, and **(D)** tumor promotion and neoangiogenesis induced by tumor-infiltrating immune cells.

Cancer-associated fibroblasts (CAFs) (5–8), myeloid-derived suppressor cells (MDSC), regulatory T cells (Tregs), type-2 macrophages (M2 macrophages), tumor-associated neutrophils (9), inhibitory cytokines, and immune checkpoint receptors are components of the immune system acting together with cancer cells, responsible for the subversion of antitumor immunity (10, 11) (Figure 1). We will discuss any of these TME components in the following sections.

### Cancer-Associated Fibroblasts

Cancer-associated fibroblasts are key players in the generation of an immunosuppressive TME and consequently in the promotion of tumor evasion from immune surveillance. It is well known that high presence of CAFs in the context of the tumor has been correlated with poor prognosis in several malignancies, including lung (12) and colorectal cancers (13). The mechanisms used by CAF to induce tumoral growth and immune escape are different: (i) the production and the release of a large amount of immunosuppressive cytokines and growth factors, such as interleukin (IL)-6 and IL-8, transforming growth factor (TGF)-β, vascular endothelial growth factor (VEGF), and insulin-like growth factor, that directly or indirectly influence the behavior of malignant cells (14) and (ii) the direct suppressor activity on T cell proliferation through regulatory molecules and immunosuppressive cytokines released by fibroblasts. In addition, the analysis of the expression of costimulatory molecules on CAF have showed that CD80 and CD86 are not expressed by CAFs or normal fibroblast while B7H1 and B7DC, that bind programmed death-1 (PD-1) on activating T cells trasducing a negative signal inside the cells, are expressed by CAFs, but not by normal fibroblasts. In non-small cell lung cancer (NSCLC) patients, Nazareth et al. have demonstrated that CAFs constitutively express the B7H1 and B7DC molecules (15) and their blocking completely restores activation of tumor-associated T cells.

Moreover, CAFs shows a direct function on the orchestration of TME, inducing preferentially T cell apoptosis and Tregs; in fact the production of IL-6, CXCL8 are responsible for tumor-associated macrophage (TAM) polarization toward M2 macrophage polarization and functions during the differentiation of circulating monocytes to TAMs (16–19) while TGF-β induce the recruitment of macrophages at the tumor site, promoting the effective tumor evasion of the host immune system (20, 21).

### Myeloid-Derived Suppressor Cells

Myeloid-derived suppressor cells play several protumoral functions promoting tumor cell survival, angiogenesis, invasion of tumor cells, and initiation of metastasis formation (22). Moreover, they can directly and indirectly skew the immune response toward immune suppressive counterparts, using different strategies:

(1) MDSCs are able to inhibit T cell proliferation and activation by (i) the depletion of essential amino acids such as l-arginine, *via* arginase-1 (ARG)-dependent consumption, and l-cystein by sequestering, as demonstrated in renal adenocarcinoma (23, 24); (ii) the reduction of local tryptophan levels and production of cytotoxic metabolites by indoleamine 2,3 dioxygenase (IDO) (25); and (iii) the decrease of IL-2 production and inhibition of the IL-2 receptor signaling, by the reactive oxygen and nitrogen species NO, ROS, H_2_O_2_, and peroxynitrite, produced by arginase 1 (Arg-1), nitric oxide synthase (iNOS), and NADPH oxidase (NOX2) (26, 27).
(2) MDSCs induce T cell apoptosis by several mechanisms, such as decrease of Bcl-2 expression and upregulation of FAS (CD95 ligand) in T cells, expression of galectin 9, which binds the inhibitory surface molecule TIM3 (T-cell immunoglobulin domain and mucin domain) and by expressing inhibitory surface molecules that alter T cell viability and trafficking.
(3) MDSCs interfere with lymphocyte trafficking and viability through the downregulation of L-selectin (CD62L) on the surface of T cells, by expression of ADAM17 (disintegrin and metalloproteinase domain 17) and they also interrupt the migration of CD8^+^ T cells to tumor sites by peroxynitrite modification of CCL2 (28, 29).
(4) MDSCs promote the differentiation of CD4^+^ T cells into Tregs both by direct cell–cell interactions (including CD40–CD40L interactions) and the production of several cytokines (such as IL-10 and TGF-β) (30), and polarize TAMs toward the M2 phenotype (31).

### Regulatory T Cells

In the TME, classic Tregs, as defined by expression of CD4, CD25, cytotoxic T lymphocyte-associated antigen-4 (CTLA-4/CD152), the Forkhead Box P3 transcription factor (32, 33), and Helios (34), directly promote immune evasion and the formation of a pro-tumorigenic TME, and prompt the growth and metastasis of various malignant tumors such as lung, ovary, breast, and prostate (35). Tregs exert their immunosuppressive activity using different approaches: they release soluble inhibitory molecules as TGF-β, IL-10, adenosine, PGE_2_, interfere with T effector cell activity and perforin/granzyme-mediated direct cytotoxicity by sequestration of IL-2 (36) and directly inhibit effector T cells by virtue of immune checkpoints and inhibitory receptors (CTLA-4, PD-1, and LAG-3) (37, 38).

### M2 Macrophages

In the TME, macrophages typically differentiate to the M2 phenotype under the action of Th2 cytokines (such as IL-4 and IL-13) and glucocorticoids. M2 macrophages promote tumor growth by suppressing immune response, remodeling the extracellular matrix, and stimulating neoangiogenesis (39). The majority of macrophages that are recruited at the tumor site, called TAMs, acquire features closely similar to the M2 phenotype due to different stimuli present in the TME, such as IL-4 and TGF-β, accompanied by reduced antitumoral activity (40). TAMs play an important role for lymphangiogenesis through the release of VEGF-C and VEGF-D *via* VEGFR3, and neo angiogenesis by VEGF, TNF-α, CXCL8, PDGF-β, MMP2, MMP7, and MMP9, both of mechanism are critical steps for tumor growth, invasion, and metastasis (41).

## Effects of the TME on γδ T Cells

γδ T cells are considered as good candidates for effective antitumor immunotherapeutical approaches for their unique features as (i) the recognition of antigens shared by a variety of stressed and tumor cells (42) in the absence of major histocompatibility complex (MHC) restriction and co-stimulation, (ii) the production of cytokines with well-known antitumor effect as IFN-γ and TNF-α with cytotoxic activity against tumor cells directly and indirectly *via* stimulating macrophages and DCs (43–45), and (iii) the potent cytotoxic activity *in vitro* and in xenograft models *in vivo* mediated by several different effector mechanisms (46–48). Moreover, γδ T lymphocytes are recruited in several types of cancer (49) and analysis of expression signatures from a large number of human tumors identified them as the most significant favorable cancer-wide prognostic signature for outcome (50, 51). Moreover, data mining transcriptomes from a large cohort of colorectal cancer patients (*n* = 585) has revealed that the aboundance of tumor-infiltrating γδ T cells is related with the 5-year disease-free survival probability (51).

There are at least three major γδ T cell subsets in humans that exhibit different Vδ chain in the TCR: (1) the population expressing the Vδ2 gene paired with the Vγ9 chain (Vγ9Vδ2 T cells) represent the majority of circulating γδ T cell population; (2) the population expressing the Vδ1 gene and different Vγ chain, are confined to skin and mucosa; and (3) a third subset of Vδ3 cells are present in higher percentage in the liver (52).

Antigen recognition by γδ T cells is a field of intense research. Vδ1 T cells recognize MHC class I-related molecule A (MICA), MHC class I-related molecule B (MICB), and UL16-binding proteins, expressed on stressed and tumor cells (53–55), glycolipid presented by MHC-related class Ib molecules CD1c and CD1d (56, 57), and unidentified ligands that engage natural cytotoxicity receptors (such as NKp30 and NKp44) (58). It is known that Vδ3 T cells can be activated by a glycolipid bound to CD1d molecules, but the real activating ligand are not yet defined (59).

Finally, Vδ2 T cells recognize non-peptidic phosphorylated intermediates of the non-mevalonate and mevalonate pathways of isoprenoid biosynthesis called phosphoantigens (PAgs), in the absence of processing, presentation, and MHC restriction (60).

Thus, there is a substantial interest in γδ T cells in the context of immunotherapeutic strategies, considering the intracellular accumulation of isopentenylpyrophosphate leading to activation of Vδ2 T cells can be manipulated in the experimental assay and applied *in vitro* and *in vivo* cancer immunotherapy by two synthetic drugs, the synthetic PAg analog bromohydrin pyrophosphate and the aminobisphosphonate (n-BP) Zoledronate.

Nonetheless, recent flow cytometry or immunohistochemical studies of tumor-infiltrating γδ T cells have failed to provide clear-cut evidence that they correlate positively or not with tumor growth, or even fail to correlate with any prognostic feature in different types of cancer, as reviewed in Ref. (61).

The dual role of Vδ2 T cells against tumor cells, either antitumoral or protumoral, could be related to the plasticity of γδ T cells to differentiate into different functional subsets under precise polarizing conditions; thus, Vδ2 T cells may display Th1-, Th2- (62), Th9- (63), Th17- (64), or Treg-like (65) profiles and they can produce several immunosuppressive cytokines as TGF-β and IL-10. Recent papers indicate that IL-17 produced by Th17-like γδ T cells can directly promote the proliferation and dissemination of tumor cells in breast cancer (66–68) and in the TME IL-17 regulates other cell population, such as MDSCs and macrophages influencing indirectly the tumor immunosurveillance (69). Treg-like Vδ2 T cells participate in the immunosuppressive TME either by the release of soluble molecules and by cell-to-cell contact *via* CD86/CTLA-4 and PD-L1/PD-1 interactions (70, 71). Recently Hu et al. have identified a novel γδ Treg subset exhibiting CD39 expression that is polarized by TGF-β, with stronger immunosuppressive potential than CD4^+^ Tregs and that suppresses the activity of human lymphocytes in an adenosine-dependent manner (72).

This plasticity of γδ T cells and the plausible idea that the TME drives their differentiation toward subsets equipped with immunosuppressive activities suggests the possibility that the TME can limit the effectiveness of the antitumor activity of γδ T cells (73).

How does then the TME induce the polarization of γδ T cells toward pro tumoral subsets?

Tumor cells and other cells of the TME produce inhibitory molecules which interfere with the proliferation and function of γδ T cells, such as TGF-β (74), prostaglandin-E2, adenosine (75, 76), and soluble NKG2D ligands (such as MICA/B) (77).

We have recently investigated the nature of the immunosuppressive soluble molecules present in secretomes from two different human cancer types. We first analyzed secretomes obtained from cancer stem cells (CSC) and CAF of non-melanoma skin cancer patients, and found that the secretome of SCC patients contains cytokines (IL-6, IL-1β, IL-23, and TGF-β) capable of polarizing the differentiation of γδ17 T cells (78, 79), confirming the transition from IFN-γ-producing to IL-17-producing γδ T cells in the TME, during tumor progression observed in these patients. Whether or not these cytokines alone are responsible for the polarization toward γδ17 T cells or additional cells/factors are required is currently under investigation: accordingly, we have recently found that activated plasmacytoid dendritic cells provide yet unknown signals which selectively induce γδ17 T cell polarization of Vγ9Vδ2 T cells (80), which was dominant over the PAg-induced IFN-γ response.

In a second study, we have also studied the immunosuppressive properties of secretomes of CAF and CSC obtained from CRC patients (51). Secretome from colon CSCs significantly inhibits proliferation and IFN-γ production by freshly γδ T cells and also by γδ, CD4^+^, and CD8^+^ αβ T cell lines and promotes production of IL-17. Conversely, secretome from CAF has limited suppressive ability and does not promote production of IL-17. Detailed analysis of CSC and CAF secretomes revealed only three cytokines differentially expressed by the inhibitory CSC secretome, but absent in the non-inhibitory CAF secretome, IL-8, IL-12, and VEGF. Because IL-12 does not have inhibitory activity on T cell proliferation and IFN-γ production, IL-8 and VEGF remain potential candidates of the immunosuppressive activities of the colon CSC secretome, which is probably not exerted directly on T cells but is rather mediated by other cell types like MDSCs, M2 macrophages, DCs, and Tregs (81, 82).

While the above findings indicate that soluble molecules present in the TME promote γδ T polarization to subvert antitumor immune response, it is likely that additional signals like prostaglandins (83), kynurenins (84), or potassium (85), are needed to achieve this effect.

Indeed, relating to the well-known ability of cancer cells to use inhibitory checkpoints to induce T cells apoptosis or anergy, Vδ2 T cells results not to be affected by this immunosuppressive mechanisms by the very low expression of PD-1 compared to conventional αβ CD8 and CD4 T cells; a recent paper have demonstrated that upon 4 days of *in vitro* stimulation by Zoledronate and IL-2, Vδ2 T cells increase the expression of PD-1 but very rapidly decrease nearly to baseline (86) as well as TIGIT that is another negative checkpoint receptor (Hayday, unpublished results). Moreover, several suppressive cells in the TME can inhibit the proliferation and cytotoxic effect of γδ T cells (87–90). For example, tumor cells promote γδ T cells polarization toward a Treg phenotype, that obstacle antitumor immunity (73), contributing to the immunosuppressive microenvironment that is characteristic of most tumor cells as breast cancer (91). Deficient γδ T cell functions have already been observed in various types of cancer, including hematological malignancies (92), liver, breast cancer (93), and HCC (94).

## Targeting TME for Therapy

Cancer immunotherapy is a highly promising new cancer treatment, that enhances the host antitumor response, increasing the number of effector immune cells, reducing host immunosuppressive mechanisms, inducing tumor killing, and modulating immune checkpoints (93).

Better knowledge on how tumor cells escape immune response has been translated into innovative therapeutic strategies that redirect immune cells to tumors and restore their cytotoxic activity against tumor cells.

γδ T cell immunotherapy, either by the use of *in vivo* expanded T cells by administration of compounds that activate them or by the adoptive transfer of *ex vivo*-activated γδ T cells, has been shown to be both feasible and safe (95).

**Figure 2:**
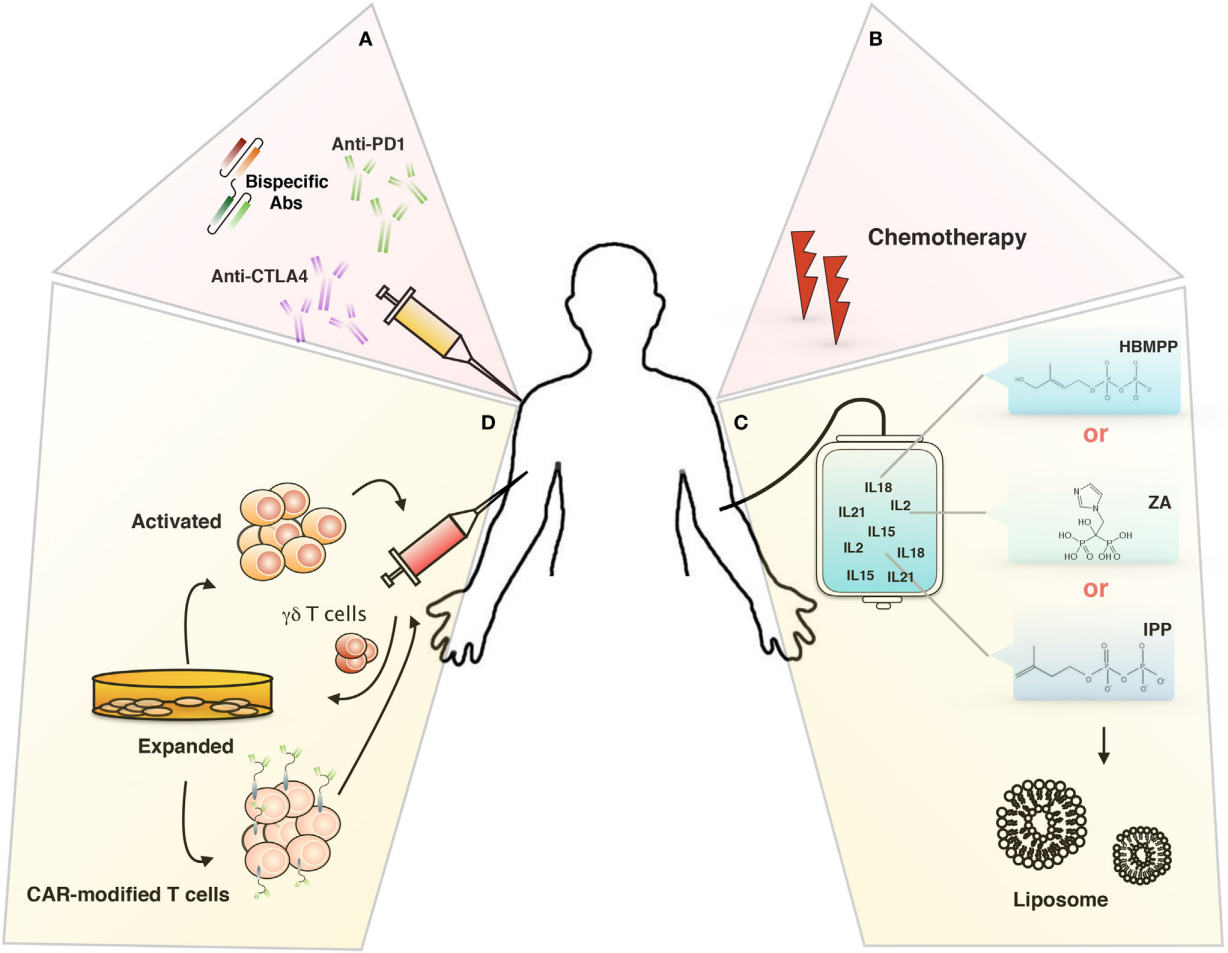
Targeting tumor microenvironment for immunotherapy. Novel therapeutical approaches to improve γδ T cells-based immunotherapy: **(A)** inhibitory immunological checkpoint and bispecific antibodies, **(B)** chemotherapy, **(C)** liposomes, and **(D)** chimeric antigen receptor-T cells (CAR-T).

However, there are technical and functional limitations to its use in cancer therapy; technical problems could be linked to the hyporesponsivness of γδ T cells in some patient or to the activation-induced γδ T cell anergy (lack of γδ T cell activation or expansion), while functional limitations could be related to the ability of *in vivo* or *in vitro*-expanded γδ T cells to reach and infiltrate tumors and to defeat the immunosuppressive environment (90, 96). To overcome this limitations, several immunotherapeutical approaches have been studied (Figure 2), we will discuss in the following sections.

### Inhibitory Immunological Checkpoint

Combination of γδ T cell adoptive transfer with immune checkpoint inhibitors is a useful strategy to enhance the antitumor activity of these cells.

The cancer immunotherapy has obtained an important progress by the use of therapeutic antibodies to CTLA-4, PD-1, and PD-L1 that antagonize immune checkpoints even though not all cancers respond to these antibody therapies. The pharmaceutical industries are involved in the development of new monoclonal antibodies that target B7H1 (PD-L1) (97, 98), considering that the blockade of B7H1 seems to induce durable tumor regression and prolonged disease stabilization in patients with advanced cancers in a phase I trial (99). Vγ9Vδ2 T cells express very low levels PD-1 compared to CD8 and CD4 T cells, with a peak at 4 days of *in vitro* activation with Zoledronate and IL-2 but it is not durable considering their rapid decrease upon 7 days reaching the baseline level [(86) and our unpublished data]. Beyond PD-1, many other immune checkpoint inhibitors as CTLA-4, IDO, VISTA, galectin-9, LAG-3, and TIM-3 should be investigated to improve γδ T cell adoptive immunotherapy.

### Chemotherapy

Some cytotoxic compounds, including doxorubicin and oxaliplatin, can trigger “immunogenic” cell death. These chemotherapeutic agents act on tumor cells in such a way that the host immune system recognizes the dying tumor cell. A tumor-specific immune response occurs during cell death, which results in an antitumor immune response leading to tumor eradication and prevention of relapse. This immunogenic cell death can prevent immune tolerance to tumor cells and is a crucial component of treatment efficacy (100). γδ T cells can be recruited to the tumor site after exposure to immunogenic chemotherapy and can contribute to its efficacy (67). *In vitro*, pre-treatment with low concentrations of chemotherapeutic agents (doxorubicin, cisplatin, etoposide, and vincristine) or even Zoledronate has been shown to sensitize tumor cells to killing by γδ T cells with additive or synergistic effects (101–104).

Therefore, such new effective immunotherapeutic approaches include the use of chemotherapeutic drugs that induce immunogenic cell death (100) or non-specific immune stimulation by cytokines such as IL-2 and IFN-α, monoclonal antibodies, and other biomolecules. Novel regimens that combine these drugs with PAgs or with γδ T cells are currently under investigation.

### Liposomes

Aminobisphosphonates (N-BPs) have been shown to have anti-cancer activity both as a monotherapy and in combination with γδ T cells. Due to the biodistribution of N-BPs *in vivo*, encapsulation of N-BPs in a nanoformulation is a good technique for their use in the treatment of non-osseous tumors.

Liposomes, a closed bilayer phospholipid system, have been proposed as drug carriers in cancer therapy due to their ability to be preferentially taken up in tumors (105), to increase the therapeutic index of a drug and to reduce the side effects (106, 107). Moreover, nanoparticles such as liposomes pass very easily trough the blood vessel, expecially the neovessels inside the tumor that exhibit leaky endothelial lining. This effect is further reinforced by the lack of efficient lymphatic drainage of the tumor which causes liposomes to accumulate preferentially in the tumor area. This is known as enhanced permeation and retention effect (EPR) (108). Particles of 10–500 nm are thought to be able to extravasate into tumors as the pore sizes in the endothelial lining of leaky blood vessels in peripheral tumors are estimated to be 400–600 nm in diameter (109). However, particles with diameters <200 nm have been shown to be more effective at accumulating at tumor sites. This passive tumor targeting does not occur in all tumors and vessel leakiness may also be heterogeneous within a single tumor (110). Ligand-targeted or “active” targeting of liposomes may result in liposomes that are more selective to cancer cells, once passive targeting has taken place (107).

Toxic side effects have been observed *in vivo* when Zoledronate and Alendronate were encapsulated into liposomes even though liposome-encapsulated (L)-Alendronate was shown to be better tolerated than L-Zoledronate. Hodgins and colleagues have obtained promising results using *in vivo* L-Alendronate and γδ T cells for the treatment of experimental metastatic lung tumors in immunocompromised mice (110).

To increase the uptake of L-Alendronate by receptor-mediated endocytosis *in vivo*, Hodgins and colleagues have targeted L-Alendronate to the α_v_β_6_ integrin receptor, which is overexpressed on cancer cells but absent on normal cells; combining the immunotherapy with γδ T cells, they achieved substantial sensitization of α_v_β_6_ positive cancer cells to γδ T cells and a more efficient cell killing *in vitro*. Despite the promise of using targeted-L-Alendronate in a monotherapy regimen, no added advantage was observed in an experimental metastatic lung mice model by the combination of targeted-L-Alendronate and γδ T cells (111).

### Bispecific Antibodies

The immunotherapeutical approaches using monoclonal antibody-based targeted therapy have obtained promising results, improved by the generation of bispecific antibody (bsAb) (112) capable of targeting multiple molecules as a single agent, even though the positive effects are not time durable because of their toxicity and cellular resistance mechanisms.

In order to be able to recruit and activate all T-cell subsets, most bsAbs target CD3, but as a consequence a wide range of T cells, including CD4^+^, CD8^+^, γδ T-cells, and also several immunoregulatory and immunosuppressive T-cell subsets are recruited.

Bispecific antibodies are very promising tools for γδ T cell-based immunotherapy with a lot advantages. There exist Vδ2γδT cell and γδ T cell (Vδ2 and Vδ1)-NK cell-specific bsAb which drastically enhance cytotoxic activity of these cells and did not recruit immunosuppressive γδ T cells (113–116).

Recently, de Bruin and colleagues produced a new bispecific nanobody that simultaneously targets Vγ9Vδ2 T cells and EGFR. This compound has shown a potent ability to activate Vγ9Vδ2 T cells and to induce their antitumoral activity *in vitro* and in mouse xenograft model *in vivo* independently on the mutational status of the tumor. Thanks to the conserved monomorphic nature of the Vγ9Vδ2 TCR that permits a more selective cell recruitment, this immunotherapeutic approach could be used in several different clinical settings and could be applied to a large group of cancer patients (117).

### Chimeric Antigen Receptor-T Cells (CAR-T)

Chimeric antigen receptors (CARs) redirect T cell specificity to tumor-associated antigens (TAAs), such as CD19, independently on the genetic (MHC) restriction.

Given the natural recruitment of γδ T cells for the tumor site, their transduction with CARs might increase their cytotoxic activity without affecting their migratory capability toward the tumor and their polarization toward antigen-presenting cells phenotypes that prolong the intratumoral immune response (118).

γδ T cells directly recognizes unique TAAs, e.g., MICA/B, F1-ATPase, and PAgs, which are widely expressed by a variety of tumor cells (119) and thus, broad recognition of tumor cells and antitumor activities may be achieved by these T cells expressing a diverse γδ TCR repertoire.

The question concerning the optimization of the immunotherapeutical approaches using costimulatory molecules remains open and the synergy between TCR γδ and costimulatory molecules signals should be better explored for clinical expansion of Vδ2 T cells.

It is well known that γδ T cells express a series of costimulatory molecules such as CD27, CD28, and 4-1BB (CD137) that increase their activation and effector function. Ribot et al. showed that CD28 is constitutively expressed on γδ T cells and play a role on the survival and proliferation *via* IL-2 production (120). deBarros and colleagues have shown the key role of CD70 molecule (CD27 ligand) on the *in vitro* expansion of Vγ9Vδ2 T cell by promoting the upregulation of Cyclin D2 and the anti-apoptotic gene regulator Bcl2a1, and on the effector function by the production of high levels of IFN-γ (121). Another costimolatory molecule investigated on Vγ9Vδ2 T cells was CD137L that is expressed at high levels when cells are activated and act as a ligand for CD137 on T and NK cells (122).

Capsomidis et al. have produced a new CAR by GD2-targeting, easily trasduced by both Vδ1 and Vδ2 subsets; the transduced cells have shown an increase cytotoxicity activity toward GD2-expressing cancer cell lines, a stable ability to migrate in tumor cells, take up tumor antigens and cross-present the processed peptide to responder αβ T lymphocytes (118).

Although these engineered immune cells have made remarkable success in the treatment of patients with hematologic malignancies, the therapeutic efficacy in solid tumors has been limited because of the complexity and the heterogeneity of TME.

## Conclusion

Recent advances in cancer immunotherapy have revolutionized treatment for a number of cancers. By targeting checkpoint receptors, durable remissions have been achieved in patients with advanced metastatic melanoma, NSCLC, bladder cancer, and kidney cancer, that otherwise would have had little chance of survival with conventional chemotherapies or targeted therapies. Similarly, CAR-T, bearing receptors specific for CD19 have successfully treated patients with relapsing B cell acute lymphoblastic leukemia and diffuse large B cell lymphoma. However, both these treatments have limitations. Therefore, additional types of immunotherapy are needed to achieve the full potential of cancer immunotherapy. Harnessing γδ T cells toward tumor cells remains a fascinating immunotherapeutical approach, considering that their activation is not dependent on peptides presented by MHC proteins and is, therefore, MHC unrestricted. Finally, the efficacy of adoptive immunotherapy with Vδ2 T cells is independent of the mutational status of the tumor (123, 124), a limit for the efficacy of checkpoint blockade, and consequently could be also applied to patients with cancers that have low numbers of mutations, such as many of the pediatric cancers.

The well-known plasticity of γδ T cells upon interaction with TME limits the effectiveness of this therapy, even though the overall interactions of the cells in TME and their rapid modifications induced by the natural story of the tumor and of the host remain an enigmatic story. A better comprehension of these mechanisms will be useful to formulate really efficient and durable therapeutic strategies that combine different approaches and could restore antitumor immune responses, overcome tumor escape, and overcome tumor-induced immune deviation to enable the host immune system to more effectively control tumor growth.

## Author Contributions

SM and FD wrote the paper. ELP prepared the figures. ELP, GP, AMC, NC, and GS contributed to the discussion of the draft.

## Conflict of Interest Statement

The authors declare that the research was conducted in the absence of any commercial or financial relationships that could be construed as a potential conflict of interest.
